# Role of Na^+ ^and Ca^2+ ^currents in computational model of *in-vitro *sigh generation

**DOI:** 10.1186/1471-2202-16-S1-P257

**Published:** 2015-12-18

**Authors:** Natalia Toporikova, Muriel Thoby-Brisson

**Affiliations:** 1 Biology Department and Neuroscience Program, Washington and Lee University, Lexington, VA, 24450, USA; 2 Institut de Neurosciences Cognitives et Intégratives d'Aquitaine, CNRS UMR 5287, Université de Bordeaux, 33076 Bordeaux, France

## 

**Figure 1:**
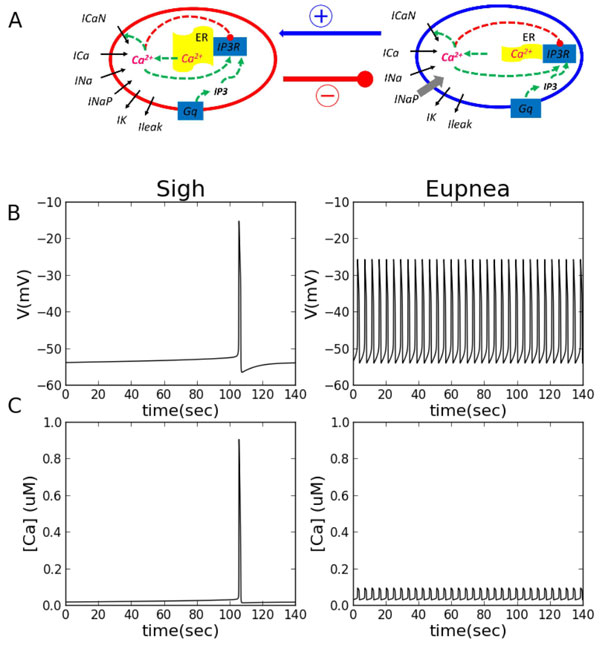
**Sigh and eupnea activity pattern *in silico***. (A) Diagrams of sigh (left) and eupnea (right) network models. Except for the ER capacity, *I_NaP _*and *I_h_*, the sigh and eupnea models are identical. (B) Intracellular *Ca^2+ ^*(top) and voltage (bottom) obtained for individual uncoupled compartments (*g_syn_*=0).

Eupneic breathing in mammals is periodically interrupted by spontaneous augmented breaths (sighs) that are characterized by a biphasic larger-amplitude inspiratory burst followed by post-sigh apnea. Previous in vitro studies in newborn rodents have demonstrated that the respiratory oscillator of the pre-Bötzinger complex (preBötC) can generate the distinct inspiratory-related motor patterns for both eupnea- and sigh-like activity [1,2]. However it remains debated whether these two types of inspiratory activities are produced by the same neuronal population or by distinct sub-networks. Based on recent in vitro data obtained in the mouse embryo [3], we have built a computational model consisting of two compartments, one dedicated to sigh generation and the other generating eupneic bursts, interconnected through appropriate synapses (Figure 1 A).

The model reproduces basic features of simultaneous sigh and eupnea generation: two types of bursts differing in terms of shape, amplitude and frequency of occurrence and mimics the effect of glycinergic synapses blockade. We designed a two-compartment computational model for sigh and eupnea subpopulations of neurons with several different parameters reflecting distinct burst generating mechanisms. The sigh subpopulation generates a low frequency rhythm based on slow intracellular Ca^2+ ^oscillations and the eupnea subnetwork generates fast oscillations mainly driven by activation/inactivation of the persistent Na^+ ^current (Fig 1 B,C).Furthermore, we used this model to make predictions that were subsequently tested on the isolated preBötC in brainstem slice preparations. Through a combination of our *in vitro *and *in silico *approaches we found that 1), sigh events are less sensitive to network excitability than eupneic activity, 2)The combination of voltage-gated calcium current and persistent sodium current control the sigh period of, and 3), specific parameters of Ih activation set the low sensitivity to excitability in the sigh neuronal subset. Altogether, our results strongly support the hypothesis that distinct subpopulations within the preBötC network are responsible for sigh and eupnea rhythmogenesis.
